# Investigation of Acetylcholine Receptor Diversity in a Nematode Parasite Leads to Characterization of Tribendimidine- and Derquantel-Sensitive nAChRs

**DOI:** 10.1371/journal.ppat.1003870

**Published:** 2014-01-30

**Authors:** Samuel K. Buxton, Claude L. Charvet, Cedric Neveu, Jacques Cabaret, Jacques Cortet, Nicolas Peineau, Melanie Abongwa, Elise Courtot, Alan P. Robertson, Richard J. Martin

**Affiliations:** 1 Department of Biomedical Sciences, College of Veterinary Medicine, Iowa State University, Ames, Iowa, United States of America; 2 INRA, UR1282 Infectiologie Animale et Santé Publique, Nouzilly, France; 3 Université François Rabelais de Tours, UMR1282 Infectiologie et Santé Publique, Tours, France; 4 Université François Rabelais de Tours, Département de Physiologie Animale, Tours, France; Free University of Berlin, Germany

## Abstract

Nicotinic acetylcholine receptors (nAChRs) of parasitic nematodes are required for body movement and are targets of important “classical” anthelmintics like levamisole and pyrantel, as well as “novel” anthelmintics like tribendimidine and derquantel. Four biophysical subtypes of nAChR have been observed electrophysiologically in body muscle of the nematode parasite *Oesophagostomum dentatum*, but their molecular basis was not understood. Additionally, loss of one of these subtypes (G 35 pS) was found to be associated with levamisole resistance. In the present study, we identified and expressed in *Xenopus* oocytes, four *O. dentatum* nAChR subunit genes, *Ode-unc-38, Ode-unc-63, Ode-unc-29 and Ode-acr-8*, to explore the origin of the receptor diversity. When different combinations of subunits were injected in *Xenopus* oocytes, we reconstituted and characterized four pharmacologically different types of nAChRs with different sensitivities to the cholinergic anthelmintics. Moreover, we demonstrate that the receptor diversity may be affected by the stoichiometric arrangement of the subunits. We show, for the first time, different combinations of subunits from a parasitic nematode that make up receptors sensitive to tribendimidine and derquantel. In addition, we report that the recombinant levamisole-sensitive receptor made up of Ode-UNC-29, Ode-UNC-63, Ode-UNC-38 and Ode-ACR-8 subunits has the same single-channel conductance, 35 pS and 2.4 ms mean open-time properties, as the levamisole-AChR (G35) subtype previously identified *in vivo*. These data highlight the flexible arrangements of the receptor subunits and their effects on sensitivity and resistance to the cholinergic anthelmintics; pyrantel, tribendimidine and/or derquantel may still be effective on levamisole-resistant worms.

## Author Summary

Parasitic nematode infections of humans and animals are world-wide. In humans, they cause disease and perpetuate a cycle of poverty. In animals, the parasites cause welfare problems and production loss. In developing countries, the debilitating effect of nematode parasites in school children limits their education, and in adults reduces productivity. These two factors, along with the production loss associated with nematode infections of animals that affects human nutrition, sustain poverty. Treatment and prophylaxis of these parasites requires the use of anthelmintic drugs. Here, we employ the *Xenopus* oocyte expression system to investigate the diversity of anthelmintic receptors in the parasitic nematode *Oesophagostomum dentatum*. We demonstrate effects of the ‘novel’ anthelmintics tribendimidine and derquantel on these reconstituted receptors, revealing, for the first time, the subunits that make up a tribendimidine- and derquantel-sensitive receptor from a parasitic nematode. We show that the receptor structure and pharmacology can be plastic, and depends on subunit composition and stoichiometry. The factors that affect the diversity of the receptor may contribute to anthelmintic resistance. Our results demonstrate the presence of acetylcholine receptor subtypes that may serve as anthelmintic targets and suggest that pyrantel, tribendimidine and/or derquantel may still be effective on levamisole-resistant worms.

## Introduction

Human nematode parasite infections are of public health concern in many developing countries where over a billion people are infected [1], [2]. Also very important for human nutrition are infections of livestock which cause significant production loss [3]. Treatment and prophylaxis for these nematode infections requires the use of anthelmintic drugs because sanitation is limited and vaccines are not available. Disturbingly, the regular use of anthelmintics has now been associated with treatment failures and gives rise to concerns about the development of resistance. Resistance has been seen, for example, against levamisole and pyrantel in animals [4]. These two ‘classic’ anthelmintics work by selectively opening ligand-gated nicotinic acetylcholine (nAChR) ion-channels of nematode muscle to produce depolarization, entry of calcium, contraction and spastic paralysis [5]. The nAChR is composed of 5 transmembrane subunits which surround the cation permeable channel pore. The importance of nAChRs has increased recently because of the introduction of novel cholinergic anthelmintics including: the agonist tribendimidine, which has been approved for human use in China [6]; the allosteric modulator monepantel [7], [8]; and the antagonist derquantel [9]. Tribendimidine is a symmetrical diamidine derivative of amidantel effective against *Ascaris*, hookworms, *Strongyloides stercolaris*, trematodes and tapeworms [10]. Molecular experiments using the free-living model nematode *C. elegans* suggest that tribendimidine acts as a cholinergic agonist like levamisole and pyrantel [11]. Derquantel is a 2-desoxo derivative of paraherquamide in the spiroindole drug class effective against various species of parasitic nematodes, particularly the trichostrongylid nematodes [12]. Derquantel is a selective competitive antagonist of nematode muscle nAChRs especially on the *B*-subtype nAChR from *Ascaris suum* [13]. In contrast to levamisole and pyrantel sensitive-nAChRs [14], [15], [16], the molecular basis for the action of tribendimidine and derquantel in parasitic nematodes are still not known. The chemical structures of the cholinergic anthelmintics are different and resistance to one of the cholinergic anthelmintics sometimes does not give rise to cross-resistance to other cholinergic anthelmintics [17]. These observations imply that the nAChR subtypes in nematode parasites may represent distinct pharmacological targets sensitive to different cholinergic anthelmintics. Therefore, in the present work, we have investigated mechanistic explanations for the variability and diversity of the cholinergic receptors of parasitic nematodes and their therapeutic significance.

The model nematode, *C. elegans*, possesses two muscle nAChR types: one sensitive to levamisole and one sensitive to nicotine [18]. The levamisole AChR type is composed of the five subunits, Cel-UNC-29, Cel-UNC-38, Cel-UNC-63, Cel-LEV-1 and Cel-LEV-8 [18], [19], [20], [21], [22]. The nicotine-AChR type is a homopentamer composed of Cel-ACR-16 subunits [23]. In *Ascaris suum*, there are three pharmacologically separate nAChR types on the body muscle: the *N*-type that is preferentially activated by nicotine; the *L*-type that is preferentially activated by levamisole and; the *B*-type that is preferentially activated by bephenium [13]. In *Oesophagostomum dentatum* there are four muscle nAChRs which are defined by their single-channel conductances: G 25 pS, G 35 pS, G 40 pS and G 45 pS. The G 35 pS receptor type of *O. dentatum* is reduced in a levamisole-resistant isolate [24] but not in a pyrantel-resistant isolate [25]. Interestingly, the G 35 pS type of *O. dentatum* has a similar channel conductance to the *L*-type from the distantly related species, *A. suum* [13].

However, the precise molecular mechanisms of levamisole and pyrantel resistance occurring in *O. dentatum* isolates are still unknown because the *O. dentatum* genes encoding muscle nAChR subunits have not yet been identified. In order to determine a mechanistic explanation for the variability and pharmacological diversity of the muscle nAChR types in parasitic nematodes like *Oesophagostomum dentatum*, we took advantage of the *Xenopus* oocyte expression system [14], [26], [27].

In this study our first objective was to clone the four putative subunit genes *Ode-unc-38, Ode-unc-29 Ode-unc-63* and *Ode-acr-8*, which are homologues of the *C. elegans* levamisole muscle receptor genes [15], [16] and express them in *Xenopus laevis* oocytes to recapitulate the levamisole-sensitive AChRs of *O. dentatum*. In a previous study on *H. contortus* which is closely related to *O. dentatum*, we have reported that co-expression of four AChR subunits (Hco-UNC-38, Hco-UNC-63, Hco-UNC-29 and Hco-ACR-8) in *Xenopus* oocytes resulted in the robust expression of a levamisole sensitive nAChR [16]. In the present work we have identified and cloned the homologs of the corresponding genes in O. dentatum (*Ode-unc-38*, *Ode-unc-63*, *Ode-unc-29* and *Ode-acr8*) and express them in Xenopus oocytes. Of particular note is the evidence that these receptor subtypes are pharmacological targets of the new anthelmintic tribendimidine. Moreover, we demonstrate that the nAChR reconstituted with all four subunits is more sensitive to levamisole than pyrantel and has a single channel conductance of 35 pS, corresponding to the *L-type* previously observed *in vivo* in *O. dentatum* and *A. suum*. Another receptor type, made of 3 subunits, was more sensitive to pyrantel and the new anthelmintic derquantel had a selective effect against receptors activated by pyrantel rather than levamisole, providing the first insight of its molecular target composition in any parasitic nematode. These results provide a basis for understanding the greater diversity of the nAChR repertoire from parasitic nematodes and for deciphering the physiological role of nAChR subtypes targeted by distinct cholinergic anthelmintics. This study will facilitate more rational use of cholinergic agonists/antagonists for the sustainable control of parasitic nematodes impacting both human and animal health.

## Results

### Identification of *unc-29, acr-8, unc-38* and *unc-63* homologues from *O. dentatum* and phylogenetic comparison to related nematode species

**Figure 1:**
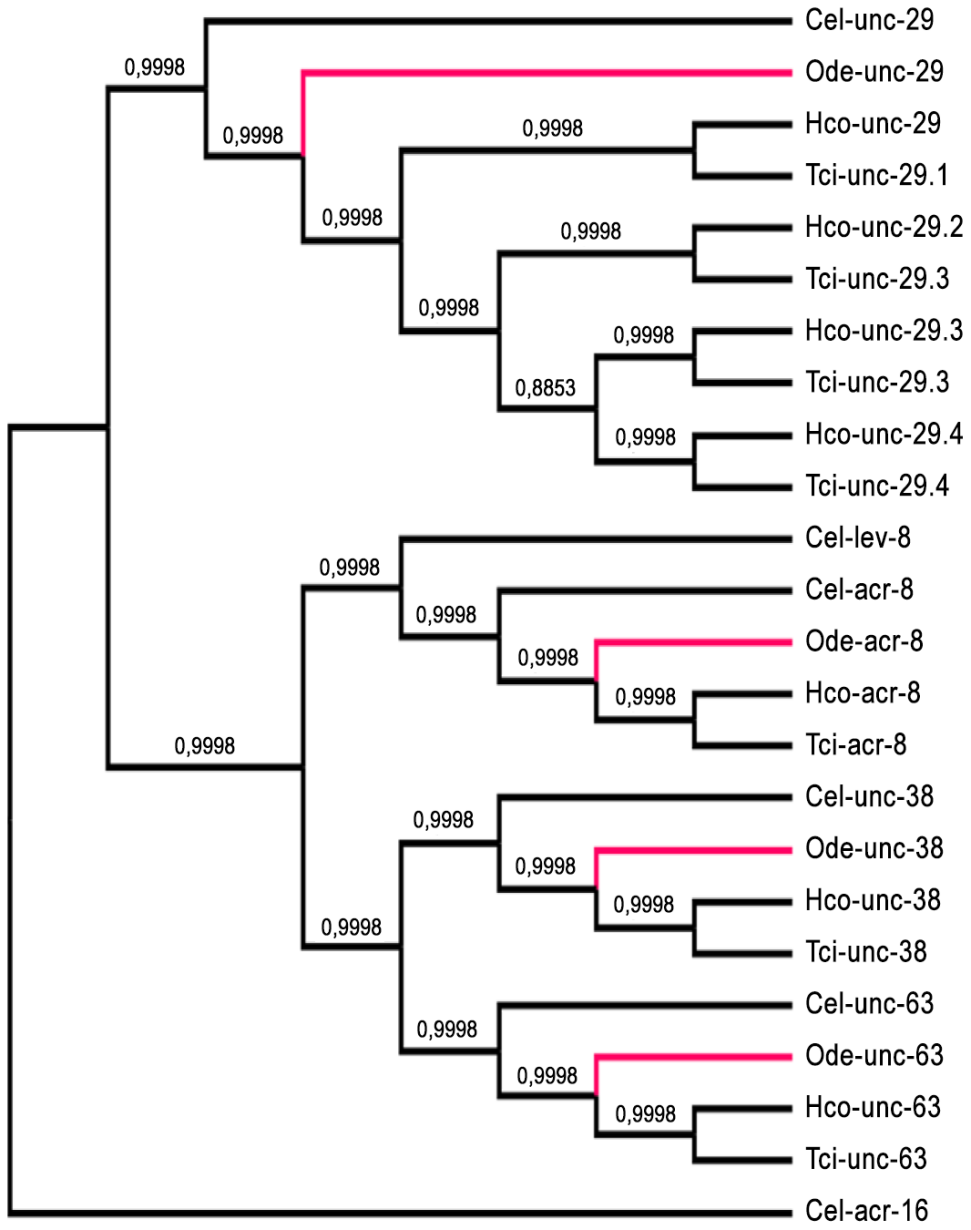
Maximum likelihood tree showing relationships of acetylcholine receptor (nAChR) subunit cDNA sequences in *Oesophagostomum dentatum* (Ode, highlighted in red), *Caenorhabditis elegans* (Cel), *Haemonchus contortus* (Hco) and *Teladorsagia circumcincta* (Tci). The *C. elegans acr-16* nAChR subunit sequence was used as an outgroup. Branch support was evaluated using the chi2 option of PhyML and values more than 0.9 were considered reliable.

Taking advantage of the phylogenetic closeness of *O. dentatum*, *C. elegans* and *H. contortus* in clade V of the nematode phylum, we used a candidate gene approach to identify full-length subunit cDNAs from *O. dentatum* of *unc-63*, *unc-29* and *acr-8* homologues. The previously available *O. dentatum unc-38*-like full-length cDNA sequence (Accession number GU256648) allowed the rapid cloning of the *Ode-unc-38* subunit cDNA. The UNC-38 and UNC-63 subunits being closely related in the UNC-38 “core” group [28], multi-alignment of *unc-63* and *unc-38* cDNA sequences from different nematode species allowed the design of degenerate primers that were able to specifically amplify the *unc-63* homologue from *O. dentatum*. To identify the *unc-29* homologue from *O. dentatum*, degenerate primers from the *H. contortus* and *C. elegans unc-29* sequences were used to amplify a partial 288 bp cDNA sequence, providing specific information to get the full-length cDNA by RACE-PCR standard procedures. To identify the *acr-8* homologue from *O. dentatum*, a partial cDNA of 861 bp was cloned using primers from *H. contortus acr-8*, allowing subsequent identification of the full-length cDNA encoding Ode-ACR-8 subunit. The four *O. dentatum* subunit transcripts were trans-spliced at their 5′end with the splice leader 1 (SL1). The predicted nAChR proteins displayed typical features of LGIC subunits including a secretion signal peptide, a cys-loop domain consisting of 2 cysteines separated by 13 amino acid residues and four transmembrane domains (TM1-4) (supplemental figure S1A–D). The Ode-UNC-38, Ode-UNC-63 and Ode-ACR-8 subunits contained in their N-terminal part the Yx(x)CC motif characteristic of the α-type subunits whereas the Ode-UNC-29 subunit sequence lacked these vicinal di-cysteines. The different nAChR subunit sequences, accession numbers, characteristics and closest levamisole subunit homologues of *C. elegans* and *H. contortus* are presented in Table 1. Alignment of the deduced amino acid sequences of the four *O. dentatum* nAChR subunits revealed 66 to 92% identity with their respective homologues from *C. elegans* and *H. contortus* (Table 1 and supplemental Fig. S1A–D), indicating that even in closely related species some interspecies polymorphisms could impact the nAChR subunit composition and pharmacological properties. The phylogenies shown in Fig. 1 (maximum likelihood tree) and supplemental Fig. S2 (distance tree) identify a single ortholog each for the *unc-38*, *unc-63*, *acr-8*, and *unc-29* subunits in *O. dentatum*. The branching topology is consistent, in each case, with a taxonomic position of *O. dentatum* with strongylida and outside the trichstrongyloidea [29]. Interestingly, this topology places the speciation of *O. dentatum* significantly before the previously identified trichostrongylid specific *unc-29* diversification event [15]. As such, these duplications cannot be shared with *O. dentatum*, although it is not possible to rule out other independent *unc-29* duplication events in *O. dentatum*. Clearly, further studies that include analysis of *unc-29* homolog sequences in a wide range of nematode species are required to understand the evolutionary impact of such duplication events.

**Table 1:** Comparison of *O. dentatum* AChR subunits with the homologs of *C. elegans* and *H. contortus*.

Gene name	Accession number	Full-length cDNA size (bp)	Deduced protein seq. length	% nucleotide identity		% amino acid identity/similarity	
				*C. elegans*	*H. contortus*	*C. elegans*	*H. contortus*
*Ode-unc-29*	JX429919	1637	497	66	73	78/87	85/90
*Ode-unc-38* a	JX429920	1681	507	66	70	72/77	83/87
*Ode-unc-63*	HQ162136	2265	507	67	80	77/84	92/95
*Ode-acr-8*	JX429921	1851	538	62	77	66/93	80/97

a Similar to complete cDNA sequence GU256648.

### Expression of *Ode(29–63)*, the *Pyr-nAChR* and stoichiometry effect on pharmacological properties

Our investigation of *O. dentatum* receptor expression determined the minimum number of subunits that is required to reconstitute a functional muscle nAChR. Previous expression studies of *C. elegans* and *H. contortus* functional nAChRs in *Xenopus* oocytes required the use of ancillary proteins [16], [26]. Hence, the *H. contortus* ancillary factors *Hco-ric-3*, *Hco-unc-50* and *Hco-unc-74* were also injected to facilitate the expression of *O. dentatum* functional receptors in *Xenopus* oocytes. In control experiments we observed that oocytes injected with ancillary protein cRNAs did not produce current responses to 100 µM acetylcholine or other cholinergic anthelmintics. We then injected different combinations of pairs of cRNAs (1∶1) of: *Ode-unc-29*, *Ode-unc-38*, *Ode-unc-63* and *Ode-acr-8*.

**Figure 2:**
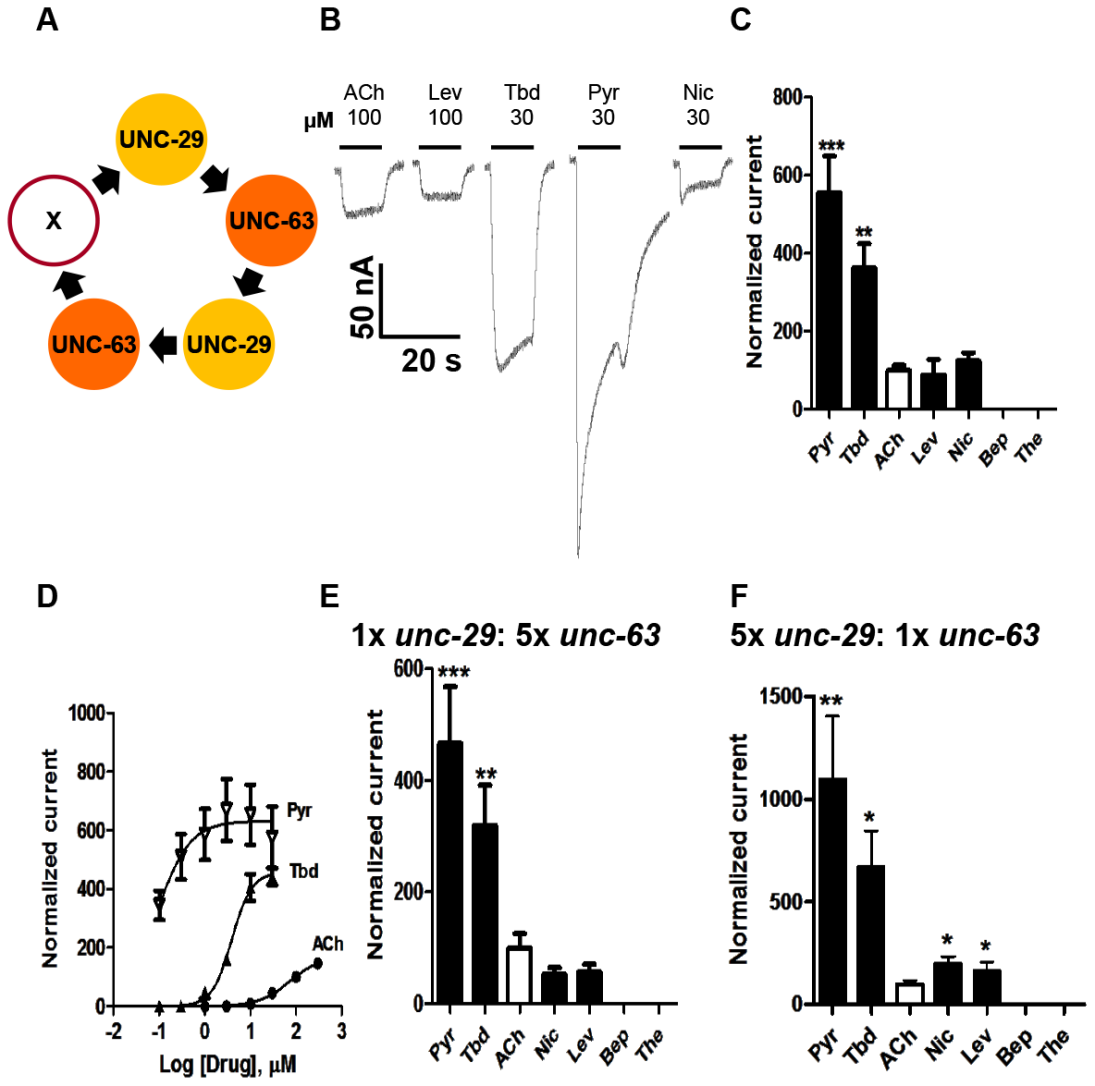
Voltage-clamp of oocytes injected with *O. dentatum Ode-unc-29* and *Ode-unc-63* nAChR subunits. (A) Diagram of possible subunit arrangements of *Ode-unc-29* and *Ode-unc-63*. X represents either UNC-63 or UNC-29 subunit. PyR, pyrantel; Tbd, tribendimidine, ACh, acetylcholine; Nic, nicotine; Bep, bephenium; The, thenium. (B) Representative traces showing the inward currents in oocytes injected with 1∶1 *Ode-unc-29* and *Ode-unc-63*. (C) Bar chart (mean ± se) of agonists-elicited currents in the *Ode*-(*29 - 63*) Pyr-nAChR, (p*aired t-test*, **p<0.01, ***p<0.001). All agonist responses have been normalized to the average 100 µM ACh currents. (D) Dose-response relationships for Pyr (inverted Δ, n = 6), Tbd (▴, n = 5) and ACh (•, n = 6) in the *Ode*-(*29 - 63*) Pyr-nAChR, (n = number of oocytes). (E) Bar chart (mean ± se) of normalized currents elicited by different agonists in 1∶5 *Ode-unc-29∶Ode-unc-63* injected oocytes. Currents have been normalized to and compared with 100 µM ACh currents (*paired t-test*, **p<0.01, ***p<0.001). (F) Bar chart (mean ± se) of normalized currents elicited by different agonists in oocytes injected with 5∶1 *Ode-unc-29∶Ode-unc-63*. Currents normalized to and compared with 100 µM ACh currents (*paired t-test*, *p<0.05, **p<0.01).

Only those oocytes injected with 1∶1 *Ode-unc29∶Ode-unc-63* cRNA, regularly produced currents of >50 nA in response to 100 µM acetylcholine or cholinergic anthelmintics, indicating that the two subunits could form functional receptors (Fig. 2). We found that no other paired combination produced currents. Without both subunits in any of our subsequent injected mixes, no functional receptor could be reconstituted, demonstrating that Ode-UNC-29 and Ode-UNC-63 are essential *O. dentatum* levamisole receptor subunits. Injection of each of these subunits alone did not form receptors that responded to the agonists we tested, demonstrating that these subunits do not form homopentamers.

More than 75% of injected oocytes responded to acetylcholine. For our standard test concentrations, except for 100 µM acetylcholine and 100 µM levamisole, we used 30 µM drug concentrations of different important anthelmintics to limit effects of open-channel block. Interestingly, the novel anthelmintic tribendimidine elicited currents when perfused on oocytes expressing Ode-UNC-29 and Ode-UNC-63. The potency series of the agonists (anthelmintics) based on the normalized currents, Fig. 2C, was: 30 µM pyrantel >30 µM tribendimidine >30 µM nicotine ≈100 µM levamisole ≈100 µM acetylcholine. Neither 30 µM bephenium, nor 30 µM thenium, were active. Even at 30 µM, pyrantel produced currents bigger than 100 µM acetylcholine (Fig. 2C). We refer to this receptor type as: *Ode*(*29–63*), or the *Pyr-nAChR*.

We determined the concentration current-response plot for pyrantel, tribendimidine and acetylcholine, Fig. 2D for *Ode*(*29–63*). The *EC_50_* values for pyrantel and tribendimidine were less than the *EC_50_* for acetylcholine but the *I_max_* values were larger for pyrantel and tribendimidine (Table 2). Thus the anthelmintics pyrantel and tribendimidine were much more potent and produced a greater maximum response than the natural ligand, acetylcholine and levamisole on this type of receptor. To date, this result represents the first characterization of a nematode tribendimidine-sensitive nAChR.

**Table 2:** Properties of the four main receptor subtypes obtained from dose-response relationships.

		*Ode*(*29-63*)	*Ode*(*29-63-38*)	*Ode*(*29-63-8*)	*Ode*(*29-63-8-38*)
Acetylcholine	*EC_50_*	72.4±13.3	13.2±0.8	3.5±0.2	4.2±0.2
	*I_max_*	100.0	100.0	100.0	100.0
	*nH*	1.28±0.31	1.10±0.044	1.18±0.055	0.77±0.06
Levamisole	*EC_50_*	*	*	2.2±0.2	3.1±2.2
	*I_max_*	*	*	73.0±2.4	119.0±3.7
	*nH*	*	*	1.36±0.18	0.87±0.10
Pyrantel	*EC_50_*	0.09±0.005	0.4±0.1	ND	ND
	*I_max_*	632.0±55.8	172.0±10.4	ND	ND
	*nH*	1.20±0.95	0.88±0.18	ND	ND
Tribendimidine	*EC_50_*	3.9±0.8	2.2±0.5	0.8±0.1	0.3±0.6
	*I_max_*	458.0±28.8	155.0±8.8	75.0±6.2	69.0±5.0
	*nH*	1.98±0.46	1.17±0.18	0.94±0.22	0.50±0.09

ND: Not determined.

* Dose-response measurements not possible due to the small size of currents elicited by the agonists on these receptor subtypes.

We were interested to see if there was evidence of pharmacological changes associated with variation in stoichiometry of the *Ode*(*29-63*) receptor. When we injected a 1∶5 ratio of *Ode-unc-29∶Ode-unc-63* cRNA with the same 1∶1∶1 ratio of ancillary proteins, the mean amplitudes of the pyrantel and tribendimidine currents were significantly increased (pyrantel: 6-fold increase from a mean of 104 nA to 653 nA, p<0.01; tribendimidine: 7-fold increase from a mean of 68 nA to a mean of 447 nA, p<0.01). The large increase in the size of the currents with increased *Ode-unc-63* cRNA suggests that increased UNC-63 favors expression of receptors with a stoichiometry of (UNC-63)_3_∶ (UNC-29)_2_ [14]. Also, when we injected 5∶1 *Ode-29*∶*Ode-63* cRNA, the normalized currents of 30 µM nicotine and 100 µM levamisole became significantly bigger than 100 µM acetylcholine (Fig. 2C, E &F) suggesting increased expression of (UNC-63)_2_∶ (UNC-29)_3_. The increase in currents with the 1∶5 and 5∶1 *Ode-29*∶*Ode-63* cRNA was not due to variability in currents with different batches of oocytes because we observed the same effects when we injected 1∶1, 1∶5 and 5∶1 *Ode-29*∶*Ode-63* cRNA in the same batch of oocytes.

### Expression of *Ode(29–63–38)*, the *Pyr/Tbd-nAChR*

**Figure 3:**
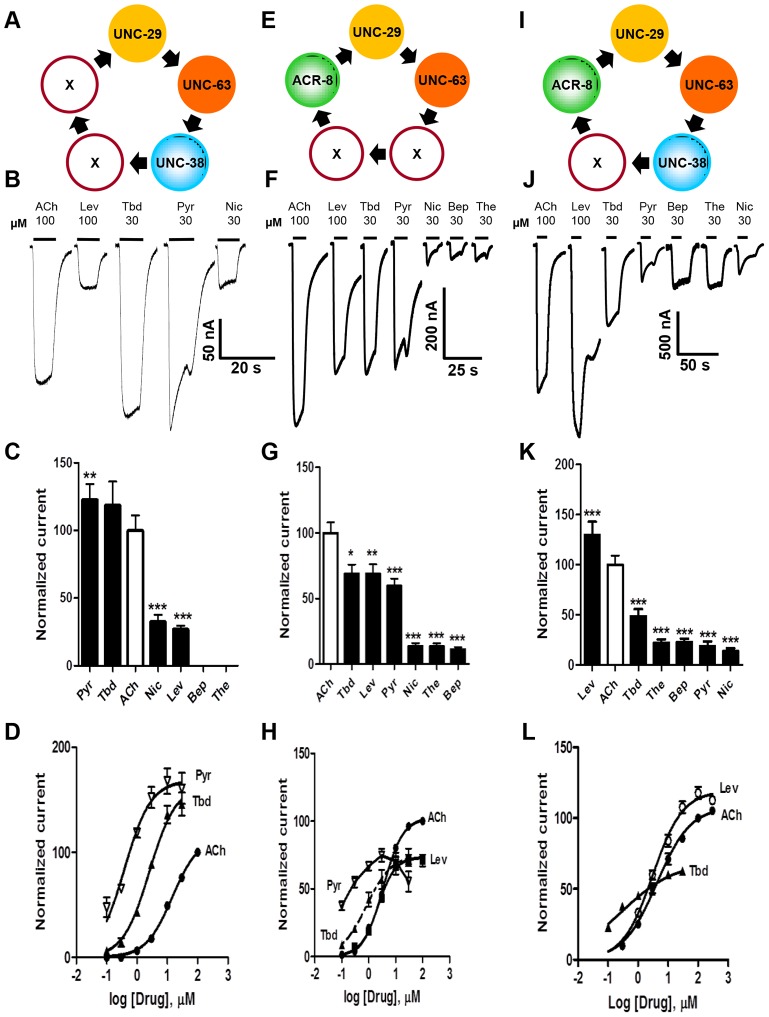
Voltage-clamp of oocytes injected with different combinations of the four *O. dentatum* nAChR subunits. (A) Depiction of a possible arrangement of *O. dentatum* UNC-29, UNC-63 & UNC-38. ‘X’ represents any of the subunits. PyR, pyrantel; Tbd, tribendimidine, ACh, acetylcholine; Nic, nicotine; Bep, bephenium; The, thenium. (B) Representative traces of inward currents elicited by the various agonists in oocytes injected with 1∶1∶1 *Ode-unc-29∶Ode-unc-63∶Ode-unc-38*. Pyr & Tbd were the most potent agonists on this receptor subtype. (C) Bar chart (mean ± se) of currents elicited by the different agonists in the *Ode*-(*29 - 38 - 63*) Pyr/Tbd-nAChR (*paired t-test*, **p<0.01, ***p<0.001). (D) Dose-response of normalized currents *vs* log concentration for Pyr (inverted Δ, n = 6), Tbd (▴, n = 6) and ACh (•, n = 5) in the Pyr/Tbd-nAChR. (E) Diagrammatic representation of the three subunits, *unc-29, unc-63* and *acr-8* injected into oocytes. ‘X’ could be any of the three subunits. (F) Representative traces of inward currents produced by the different agonists in oocytes injected with 1∶1∶1 *Ode-unc-29∶Ode-unc-63∶Ode-acr-8*. (G) Bar chart (mean ± se) of normalized currents elicited by the different agonists in the *Ode*(*29-63-8*) receptor subtype. All currents normalized to 100 µM ACh currents; comparisons made with the ACh currents (*paired t-test*, *p<0.05, **p<0.01, ***p<0.001). (H) Dose-response plot of normalized currents vs log. Concentration of Pyr (inverted Δ, n = 6), Tbd (▴, n = 6), ACh (•, n = 6) and Lev (▪, n = 6). (I) Representation of a possible arrangement of the four *O. dentatum* subunits injected into *Xenopus* oocytes. (J) Representative traces of inward currents elicited by the different agonists on the *Ode*(*29–63–38–8)* or Lev-nAChR. (K) Bar chart (mean ± se) of normalized currents elicited by the different agonists on the Lev-nAChR subtype. Comparisons were made with 100 µM ACh currents, which was used for the normalization (*paired t-test*, ***p<0.001). (L) Dose-response plot of normalized currents against log of Tbd (▴, n = 6), ACh (•, n = 18) and Lev (○, n = 5) concentrations.

We subsequently added *unc-38* and injected *Ode-unc-29∶Ode-unc-63∶Ode-unc-38* cRNAs in the ratio 1∶1∶1, along with the three *H. contortus* ancillary factors. Oocytes injected with this mix responded with larger currents of around 250 nA to 100 µM acetylcholine and to all the other agonists tested except bephenium and thenium (Fig. 3A–D). Pyrantel was the most potent agonist but with this receptor type, pyrantel and tribendimidine produced the same amplitude of current at 30 µM. We termed this receptor the *Ode*(*29–63–38*), or the *Pyr/Tbd-nAChR*. The potency series of the agonists based on the normalized currents was 30 µM pyrantel ≈30 µM tribendimidine >100 µM acetylcholine >30 µM nicotine ≈100 µM levamisole (Fig. 3 C). The dose-response curves for pyrantel, tribendimidine and acetylcholine are shown in Fig. 3D. The *EC_50_* for pyrantel was less than the *EC_50_* for tribendimidine and acetylcholine (Table 2). The Hill slopes for tribendimidine and acetylcholine were both close to 1.0 (Table 2), showing little co-operativity between agonist concentration and response. Again interestingly, the two anthelmintics, pyrantel and tribendimidine were more potent than the natural ligand and the anthelmintic levamisole, but with this receptor type, the currents were larger than with the *Ode*(*29–63*) receptor.

### Expression of *Ode*(*29–63–8*), the *ACh-nAChR*

When we added *Ode-acr-8* and injected *Ode-unc-29∶Ode-unc-63∶Ode-acr-8* cRNA in the ratio 1∶1∶1 along with the three *H. contortus* ancillary factors into the oocytes, we observed even bigger receptor responses to 100 µM acetylcholine. We also observed responses to all the agonists we tested, including bephenium and thenium (Fig. 3E–H), which suggests that *Ode-acr-8* introduces bephenium and thenium binding sites. 100 µM acetylcholine produced the largest current responses, always greater than 800 nA and sometimes greater than 1 µA. We termed this receptor type: *Ode*(*29–63–8*), or the ACh-nAChR.

For the *Ode*(*29–63–8*) receptor, the potency series of the agonists (anthelmintics) based on the normalized currents, was 100 µM acetylcholine >30 µM tribendimidine ≈100 µM levamisole ≈30 µM pyrantel >30 µM nicotine ≈30 µM thenium ≈30 µM bephenium (Fig. 3G). Here, the least potent agonists were nicotine, bephenium and thenium with average currents <20% of the acetylcholine currents. The dose-response curves (Fig. 3H) show that at the highest concentrations tested, levamisole and tribendimidine current responses were less than that of acetylcholine. Despite this, the *EC_50_* values of both tribendimidine and levamisole were lower than acetylcholine on this receptor type. The *EC_50_* values are summarized in Table 2. The levels of co-operativity (slope) were similar for these three agonists, but *I_max_* for acetylcholine (100.0%) was bigger than *I_max_* for tribendimidine (75.0±6.2%) and levamisole (73.0±2.4%).

Two characteristic features of the response of the *Ode*(*29–63–8*) receptor to pyrantel suggest the presence of open-channel block which is more pronounced with *Ode*(*29–63–8*) than with the other types. Firstly, the maintained application of 30 µM pyrantel produced a response that declined rapidly despite the concentration being maintained, and secondly, on wash-out of pyrantel the response ‘rebounded’ or increased temporally before declining. Pronounced open-channel block with pyrantel has been observed at the single-channel level [30]. Altogether, these results highlight for the first time the reconstitution of a new functional receptor with distinct pharmacological profile made of the UNC-29, UNC-63 and ACR-8 subunits.

### Expression of *Ode*(*29–63–8–38*), the *Lev-nAChR*

Finally, we injected all our subunits in a mix of *Ode*-*unc-29∶Ode*-*unc-63∶Ode*-*acr-8∶Ode*-*unc-38* cRNA in the ratio 1∶1∶1∶1 along with the three *H. contortus* ancillary factors. These subunits reconstituted a receptor type on which 100 µM levamisole produced the biggest responses of >1.5 µA, sometimes reaching >4.5 µA, Fig. 3I–L. We termed this receptor type: *Ode*(*29–63–8–38*) or the *Lev-nAChR*. The potency series of the agonists based on the normalized currents, was 100 µM levamisole >100 µM acetylcholine >30 µM tribendimidine >30 µM thenium ≈30 µM bephenium ≈30 µM pyrantel >30 µM nicotine (Fig. 3K). Here, the least potent agonists were thenium, bephenium, pyrantel and nicotine with average currents <25% of the acetylcholine currents. The *EC_50_* for tribendimidine was less than the *EC_50_* for levamisole and acetylcholine (Table 2). The dose-response curves were rather shallow with Hill slopes less than one for acetylcholine, levamisole and tribendimidine (Table 2). We have shown that expression of Ode-UNC-29, Ode-UNC-63 Ode-ACR-8 and Ode-UNC-38 produced large currents, and that the receptor, *Ode(29–63–8–38)*, was most sensitive to levamisole. It is clear from the observations above that each of the four subunits: Ode-UNC-29, Ode-UNC-63 Ode-ACR-8, and Ode-UNC-38 may combine and be adjacent to different subunits as they form different pentameric nAChRs.

### Antagonistic effects of derquantel

**Figure 4:**
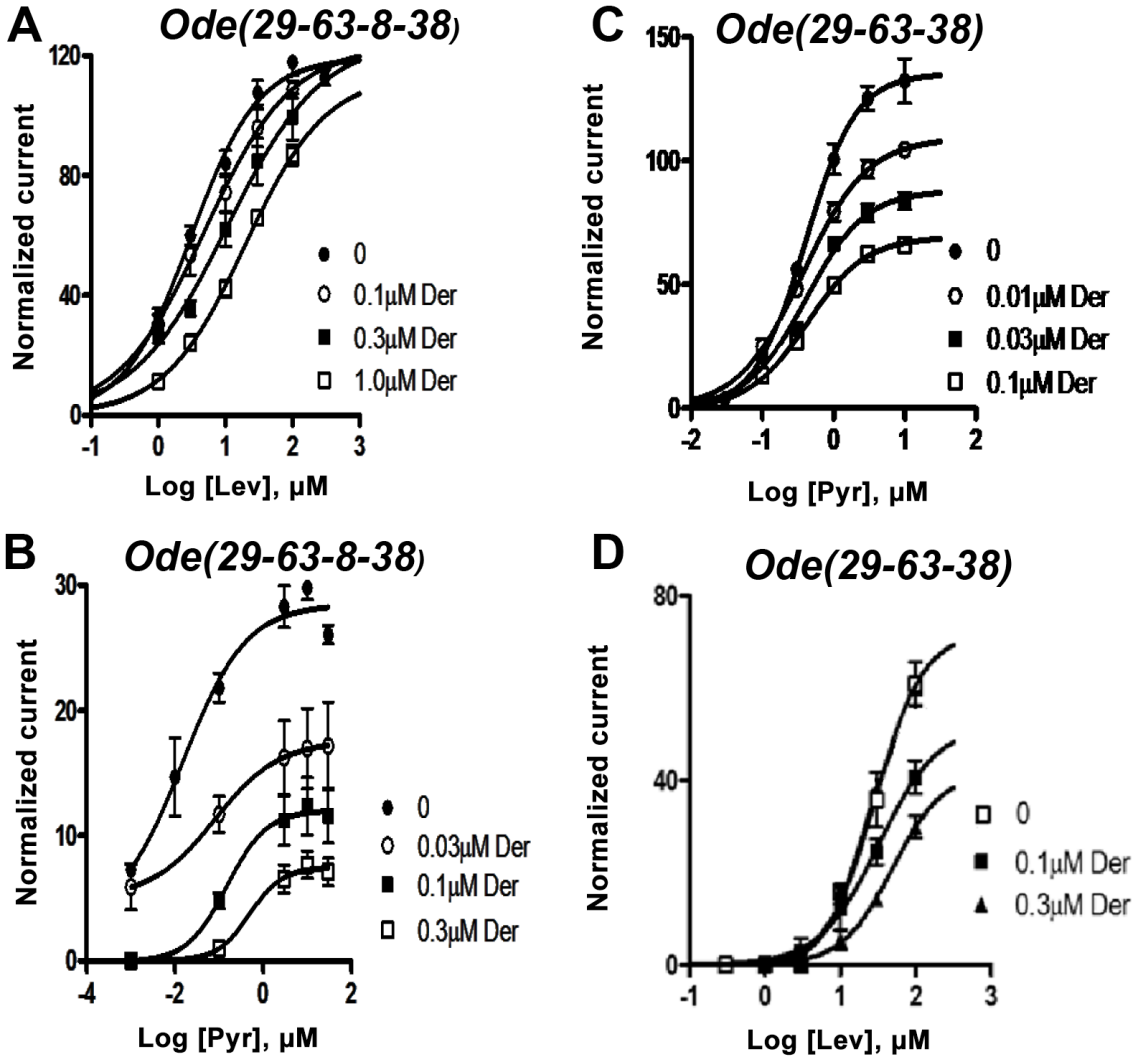
Effects of derquantel on levamisole-activated and pyrantel-activated expressed *Ode(29-63-8-38), the Lev-nAChR*, and on pyrantel-activated and levamisole-activated *Ode(29-63-38), the PyR/Trbd-nAChR* subtypes. Der: derquantel; Lev: levamisole; Pyr: pyrantel. (A) Antagonistic effects of varying derquantel concentrations on levamisole currents of *Ode(29-63-8-38)*. Levamisole evokes supramaximal normalized currents and derquantel competitively inhibited levamisole currents. (B) Derquantel antagonism of pyrantel currents of *Ode(29-63-8-38)*. Here, derquantel produced mixed non-competitively competitive antagonism. Pyrantel did not activate supramaximal currents. (C) Antagonism of pyrantel by derquantel on the *Ode(29-63-38*), Pyr/Tbd-nAChR. Derquantel is a potent non-competitive antagonist. (D) Derquantel non-competitively antagonized levamisole responses on the *Ode(29-63-38*) receptor.

We tested the antagonist effects of derquantel on the levamisole and pyrantel concentration-response plots of *Ode (29-63-38-8)* and *Ode (39-63-38)*, Fig. 4A–D. Recall that levamisole is more potent as an agonist on *Ode(29–63–8–*38) and pyrantel is more potent on *Ode(39-63-38)*. Fig. 4A shows that derquantel behaves like a potent competitive antagonist of levamisole, with 0.1 µM producing a right shift in *EC_50_* dose-ratio of 1.7 (the antagonist negative log dissociation constant from the Schild equation, *pK_B_*, was 6.8±0.1). When we tested the antagonist effects of derquantel on *Ode(29–63–8–38)* using pyrantel as the agonist, we saw mixed non-competitive and competitive antagonism: there was a right-shift in the *EC_50_* and a reduction to 41% of the maximum response by 0.1 µM derquantel, Fig. 4 B. One explanation for these mixed effects of derquantel is that co-expression of UNC-29, UNC-63, UNC-38, ACR-8 subunits produces a mixture of receptors: mostly *Ode(39-63-38-8)* receptors that are preferentially activated by levamisole and which are competitively antagonized by derquantel and; a smaller number of receptors like *Ode(39-63-38)* that are preferentially activated by pyrantel and antagonized non-competitively by derquantel. To test this further we examined the antagonist effects of derquantel on pyrantel and levamisole activation of *Ode (39-63-38)*. Derquantel antagonized both agonists non-competitively, Fig. 4 C & D. We interpret these observations to suggest that expression of *Ode(39-63-38-8)* receptors can give rise to expression of a smaller number of other receptors including *Ode(39-63-38)*. We comment further on these observations in our discussion. Nonetheless, the competitive and non-competitive antagonism of derquantel when levamisole is used on the two different receptor subtypes, Fig. 4 A & D, illustrates that derquantel effects are subtype selective.

### The receptor types have different calcium permeabilities

The *EC_50_* concentrations of acetylcholine for the different expressed receptors varied (Table 2), suggesting that there are different physiological functions for the different nAChR types. One physiological difference may be the permeability to the second messenger, calcium. To examine this we measured the relative permeability of calcium in *Ode*(*29–63–38*), *Ode*(*29–63–8*) and *Ode(29–63–8–38)*, receptors which produced large enough currents to determine reversal potentials.

**Figure 5:**
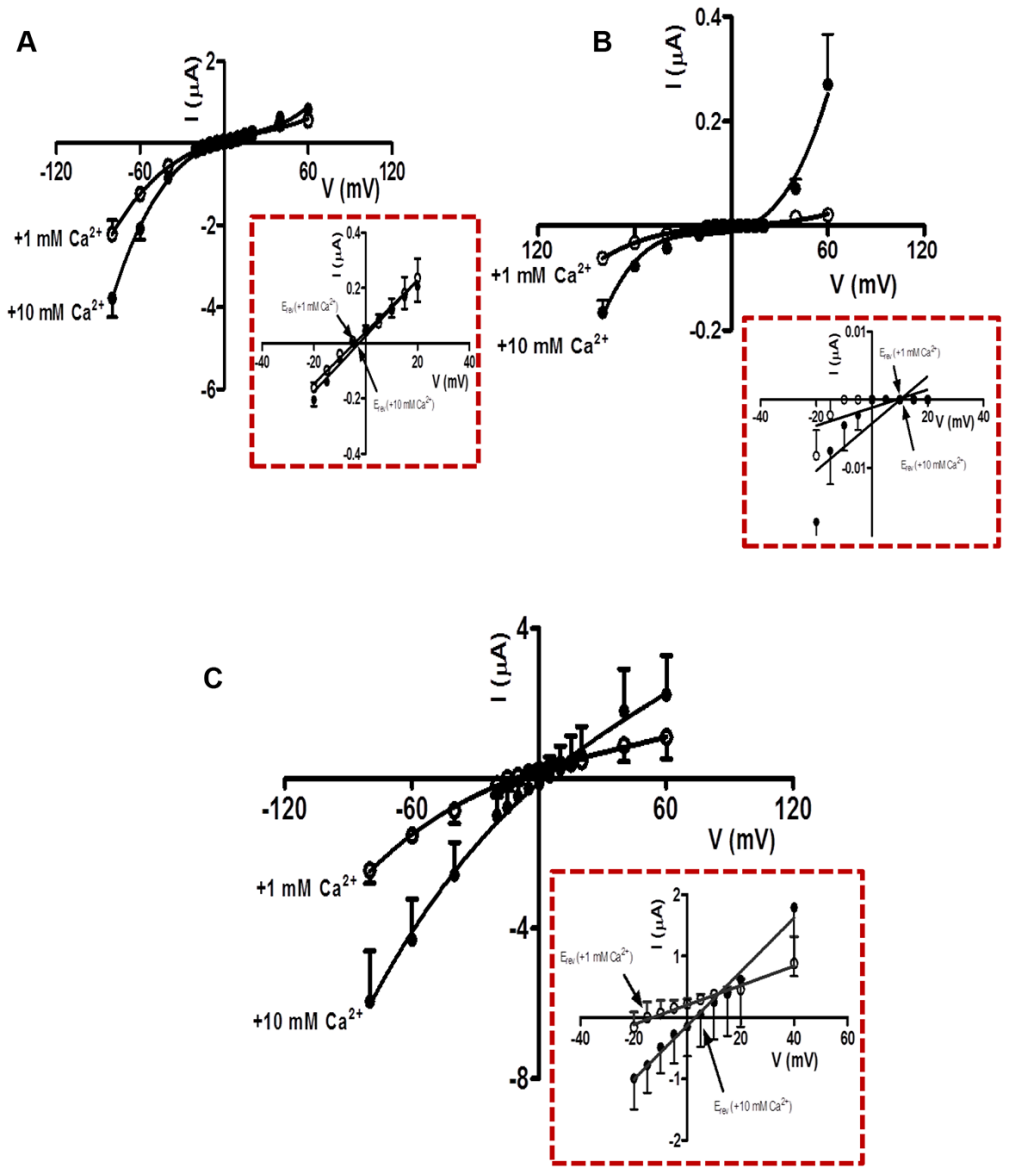
Ca^2+^ permeability of the *O. dentatum* receptor subtypes with large ACh currents. (A) Current-Voltage plot for oocytes injected with *Ode*(*29–63–8)*, showing the change in current with voltage in 1 mM and 10 mM Ca^2+^ recording solutions. *Insert:* Magnified view of current-voltage plot from −20 mV to +40 mV showing the *E_rev_* in 1 mM and 10 mM extracellular Ca^2+^. (B) Current-Voltage plot for oocytes injected with *Ode*(*29–63–38)* showing the current changes in 1 mM and 10 mM Ca^2+^ recording solution under different voltages. *Insert:* Magnified view of current-voltage plot from −20 mV to +40 mV showing the *E_rev_* in 1 mM and 10 mM extracellular Ca^2+^. (C) Current-Voltage plot for oocytes injected with *Ode*(*29–63–38–8)* in 1 mM and 10 mM Ca^2+^ recording solutions. *Insert:* Magnified view of current-voltage plot from −20 mV to +40 mV showing the *E_rev_* in 1 mM and 10 mM extracellular Ca^2+^. The calcium permeability was calculated using the GHK equation (Text S1 Legend).

We observed two different effects of increasing calcium from 1 mM to 10 mM on the acetylcholine currents. One effect was potentiation of the current amplitudes, an effect that was voltage-dependent (Fig. 5A, B & C). The voltage-dependent increases in acetylcholine currents was prominent in *Ode(29–63–8–38)*, and less in the other two receptor types. The potentiating effect of calcium on the acetylcholine currents indicates the presence of positive allosteric binding sites on one or more subunits of the pentameric nAChR [31]. The second effect was a positive shift to the right of the current-voltage plot indicating that the channels are permeable to calcium [32]. A shift in reversal potential of 1.7 mV was recorded for the *Ode*(*29–63–8*) (Fig. 5A). Using the Goldman Hodgkin Katz constant field equation (Text S1 Legend), we calculated this change in reversal potential corresponds to a relative calcium permeability ratio, *P_Ca_/P_Na_* of 0.5. The reversal potential shift for *Ode*(*29–63–38*) was similar, 1.3 mV, also giving a permeability ratio *P_Ca_/P_Na_* of 0.4 (Fig. 5B). For *Ode(29–63–8–38)*, the shift was 16.0 mV (Fig. 5C), corresponding to a calcium permeability ratio, *P_Ca_/P_Na_*, of 10.3. The *Ode(29–63–8–38)* receptor, was therefore much more permeable to extracellular calcium and positively allosterically [31] modulated by extracellular calcium than the other two receptor types, a physiologically significant observation.

### *Ode(29–63–8–38)*, the *Lev-nAChR* channels have a mean conductance of 35 pS

**Figure 6:**
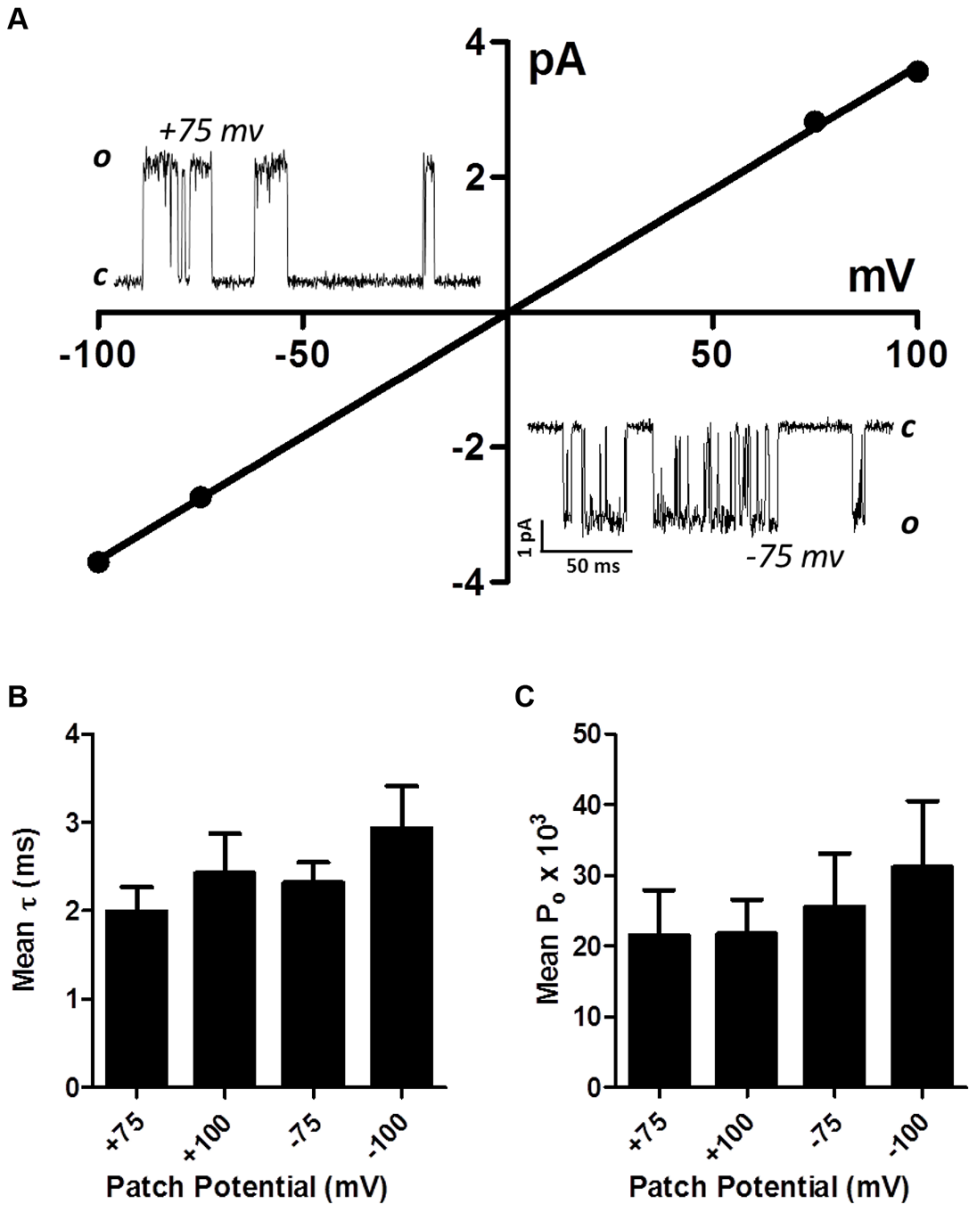
Single-channel properties of the *Lev-nAChR* subtype. (A) Representative current-voltage plot from oocyte-attached patch with 10 µM levamisole. Inserts are representative channel openings at +75 mV and −75 mV membrane potentials. Even with 10 µM levamisole, we sometimes observed ‘flickering’ channel block events; note the ‘flickering’ channel block shown by openings at −75 mV. (B) Bar chart (mean ± se) of the mean open times (τ) at the different patch potentials. (C) Bar chart (mean ± se) of the probability of channel opening, *P_o_*, at the different patch potentials.

Single-channel studies in *Oesophagostomum dentatum* body muscle have demonstrated the presence of different nAChR types with conductances of 25, 35, 40 and 45 pS, showing the presence of four or more receptor types. We have shown in experiments here, that combinations of four nAChR subunits from this parasite can produce four pharmacologically different receptor types. We found that the expressed *Ode*(*29–63–38–8*) *Lev-nAChR* type was most sensitive to levamisole and had a high permeability to calcium and hypothesized that this type may be the G35 type. Accordingly we investigated the single-channel properties of this type under patch-clamp using 10 µM levamisole as the agonist. In control oocytes injected with only the ancillary proteins, we recorded no nAChR-like channel currents in 4 oocytes. We found however, that in patches from oocytes expressing the *Ode(29–63–8–38)* type, that more than 90% of patches contained active, recognizable nAChR channels with one main conductance level. Fig. 6A shows representative channel currents from one of these patches and its current-voltage plots which had a conductance of 36.6±0.5 pS (mean ± SE). The mean conductance of channel from patches made from five different oocytes was 35.1±2.4 pS. We did not test the effects of different cholinergic anthelmintics on channel conductance. The reversal potentials of all plots were close to 0 mV, an indication that the channel was nonselective and permeable to Cs^+^ (reversal potential of 0 mV).

We measured the mean open times, *τ*, at the different potentials and observed values of 2.4±0.4 ms at +100 mV and 2.9±0.5 ms at −100 mV (Fig. 6B). The mean open times are comparable to the mean open times recorded *in vivo* in *O. dentatum* with the same concentration of levamisole.

### The three ancillary proteins affect expression levels

We tested the requirements for the ancillary proteins, RIC-3, UNC-50 and UNC-74, by removing each of the ancillary proteins in turn when expressing the *Ode(29–63–8–38)* receptor. We found that the requirement for all the ancillary proteins was not essential and that each protein, when absent reduced currents produced by the expressed receptors (Fig. S3). When all the ancillary factors were removed, we observed no measurable currents in response to acetylcholine. Here, we have observed that unlike in *C. elegans*, robust functional *O. dentatum* receptors can be reconstituted when one of these three ancillary factors is omitted and that the ancillary proteins can affect the balance of the pharmacology of the receptors expressed (Fig. S3)

## Discussion

### The origin of diverse nAChR types in parasitic nematodes

We have shown previously in the parasitic nematodes, *O. dentatum* [24] and *A. suum* [13], that there are diverse types of nAChR on body muscle of parasitic nematodes which may be activated by cholinergic anthelmintics. In *A. suum* we found that these types have different sensitivities to different cholinergic anthelmintics like levamisole and derquantel. In *O. dentatum* there are four types that can be distinguished by their single-channel conductance, with reduced numbers of the G 35 pS type in levamisole resistant isolates. Here we explored and tested the hypothesis that the receptor subtypes may be due to different combinations of subunits. We found that four main pharmacology types could be produced and separated by expressing in *Xenopus* oocytes different combinations of the α-subunits Ode-UNC-38, Ode-UNC-63 and Ode-ACR-8 together with the non-α subunit Ode-UNC-29. The types could be separated on the basis of their *EC_50_* and amplitude of response to the natural ligand acetylcholine, permeability to calcium and anthelmintic profiles (rank order potency and/or concentration-response relationships). We have not tested the possibility that post-translational modification may serve as an additional mechanism to produce different receptor types.

### The different AChR types have different functional properties

*Ode*(*29–63–38–8)*, *Ode(29–63–8)* and *Ode*(*29–63–38*) produce the largest current responses to acetylcholine. On the basis of the larger size of the currents responses to acetylcholine and the smaller currents of *Ode*(*29–63*) mixture, we speculate that the other three receptor types have more important physiological roles in the worm. *Ode*(*29–63–38*) was the most sensitive type to acetylcholine with an *EC_50_* of 3.5±0.2 µM but it had a lower calcium permeability, *P_Ca_/P_Na_* of 0.5, than *Ode*(*29–63–38–8*) that had an acetylcholine *EC_50_* of 4.2±0.2 µM. However, *Ode*(*29–63–38–8*) had greater responses to levamisole and a calcium permeability, *P_Ca_/P_Na_* of 10.4. Thus the addition of ACR-8 is responsible for increasing the calcium permeability and the response to levamisole. Since the *Ode*(*29–63–38–8*) receptor produced the biggest current to levamisole and had a single-channel conductance of 35.1±2.4 pS, close to the *in vivo* G 35 pS type that is reduced with levamisole resistance, we suggest that *Ode*(*29–63–38–8*) subunit combinations give rise to the *in vivo* G 35 pS channel in *O. dentatum*. There may also be pH regulatory effects on channel activity of *Ode*(*29–63–38–8*). The presence of histidine (*pKa* 6.0) in the ACR-8 (Fig. S1D, position 282) channel pore entrance on TM2 region is predicted to produce a pH sensitive channel that is less permeable to cations and calcium at pH 6.0 than 7.5. ACR-8 may provide a functional mechanism of reducing depolarization and muscle contraction at low pHs that can occur in an anaerobic environment. It would also have been of interest to look at the single-channel properties of the other receptor types when activated with levamisole to compare with the *in vivo* channel properties. Unfortunately, the smaller size of the levamisole currents due to the presence of fewer receptors on the oocytes, made single-channel recording less tractable than for *Ode*(*29–63–38–8*).

### Different nAChR types and sensitivities to levamisole, pyrantel, tribendimine and derquantel

The different nAChR types have different sensitivities to cholinergic anthelmintics: for example *Ode*(*29–63–38–8*) produces the biggest response to levamisole, while *Ode*(*29–63–38*) produces the biggest response to pyrantel. If these two types of receptors exist together in the nematode parasite, our observations suggest that combinations of levamisole and pyrantel will produce a bigger therapeutic response than either of the two anthelmintics given alone and that combination therapy may be advantageous. However, very few reports of combining levamisole and pyrantel for treatment are available. In one report [33] using *T. muris* infected mice, combination of levamisole and pyrantel treatment produced antagonism but these authors did not determine the *EC_50_* dose for pyrantel pamoate and used a very high dose of 300 mg/Kg dose, above the normal therapeutic dose. At this level we anticipate the presence of open-channel block limiting the effects of the therapeutic combination [32].

We tested the effects of derquantel on the *Ode*(*29–63–38–8*), the *Lev-nAChR*, and on *Ode(29-63-38*), the *Pyr/Trbd-nAChR*. Interestingly, the mode of action of derquantel varied (competitive or non-competitive) with the receptor subtypes when activated by the same agonist, levamisole. The difference in the mode of action will relate to the preferred non-equivalent binding sites of derquantel, of levamisole and of pyrantel on the receptor subtypes. The competitive antagonism of levamisole suggests a common binding site for derquantel on the *Ode*(*29–63–38–8*) receptor; the non-competitive antagonism of pyrantel and levamisole by derquantel on *Ode*(*29–63–38*) suggests negative allosteric modulation and different non-equivalent binding sites for derquantel and the agonists, levamisole and derquantel, on this receptor. Interestingly the loss of the ACR-8 subunit, which reduces the levamisole sensitivity of the *Ode(29-63-38)* receptor, changes the antagonism of levamisole from competitive to non-competitive. Thus ACR-8 may contribute to a high affinity binding site of levamisole and a competitive site of derquantel. When ACR-8 was not present derquantel behaved non-competitively. We have interpreted the mixed non-competitive competitive antagonism of pyrantel by derquantel on *Ode*(*29–63–38–8*) to be due to a mixed expression of receptor subtypes with mostly the levamisole sensitive *Ode*(*29–63–38–8*) receptors being present and a smaller proportion of other receptors like *Ode(29-63-38)* being present. This is certainly possible because, as we have shown, the subunits can and do combine in different ways to form 4 types of receptor. Although less likely, it may be that: 1) expression of *Ode(29-63-38-8)* produces only one receptor type, 2) levamisole and pyrantel bind to separate sites on this receptor, 3) derquantel acts competitively, binding at the levamisole site but; 4) derquantel acts as a negative allosteric modulator when pyrantel binds to its receptor. Further study is required to elucidate details of binding sites on the different receptor types. Despite these possibilities we have seen potent antagonist effects of derquantel on both the *Ode*(*29–63–38-8*) and the *Ode*(*29–63–38*) subtypes suggesting that derquantel could still be active against levamisole resistance associated with reduced ACR-8 expression.

We were also interested in the effects of the novel cholinergic anthelmintics tribendimidine [6] and to compare their effect with the other anthelmintics. We found that tribendimidine was a potent cholinergic anthelmintic on the *Ode*(*29–63–38*), *Ode*(*29–63–8*) and *Ode*(*29–63–38–8*) types. Thus tribendimidine could remain an effective anthelmintic if levamisole resistance in parasites were associated with null mutants or decreased expression of *acr-8* and/or *unc-38*.

### Regulation of AChR type expression

The different functional properties of the nAChR types indicate that each type may have different physiological roles to play in the parasite and the proportion of the different types present at the membrane surface is likely to be regulated. This regulation could allow changes in calcium permeability, desensitization, receptor location, receptor number and anthelmintic sensitivity. The control is expected to include dynamic physiological processes and developmental process that take place over different time scales. An adaptable and diverse receptor population may allow changes, even during exposure to cholinergic anthelmintics. We know little about these processes, their time-scales and how they may be involved in anthelmintic resistance in parasitic nematodes but some information is available from *C. elegans*.

In *C. elegans* there are a number of genes involved in processing and assembly of the subunits of AChR; specific examples include *Cel-ric-3, Cel-unc-74* and *Cel-unc-50* [34], [35], [36]. Cel-RIC-3 is a small transmembrane protein, which is a chaperone promoting nAChR folding in the endoplasmic reticulum [37]. The gene *Cel-unc-74* encodes a thioredoxin-related protein required for the expression of levamisole AChR subunits [38]. The gene *Cel-unc-50* encodes a transmembrane protein in the Golgi apparatus [35]. In *Cel-unc-50* mutants, levamisole nAChR subunits are directed to lysosomes for degradation. We found that acetylcholine currents and pharmacological profile of the receptors were sensitive to the presence of the accessory proteins, RIC-3, UNC-74 & UNC-50, suggesting the possibility that the expression level of these proteins could contribute to anthelmintic resistance in parasitic nematodes.

### Species differences

We show here that four *O. dentatum* receptor subunits can be used to reconstitute different functional levamisole receptor types and that levamisole is a full agonist in one of the main types. All of the four main *O. dentatum* receptor types responded to nicotine, which supports our use of the term nAChR. In contrast, in the expressed levamisole-sensitive AChRs of *C. elegans*, levamisole is a partial agonist, requires five subunits (Cel-UNC-38:Cel-UNC-29:Cel-UNC-63:Cel-LEV-1:Cel-LEV-8) and does not respond to nicotine. The *C. elegans* nicotine-sensitive muscle receptor is a separate homopentamer of ACR-16 [39]. We also saw differences between the expressed receptors of *O. dentatum* and the expressed AChR of *H. contortus* [16]: only two types of AChRs were produced in *Xenopus* oocytes expressing *H. contortus* subunits. Furthermore, we saw differences and similarities between the expressed *O. dentatum* receptors and the expressed *A. suum* receptors [14]. Only two subunits were used to reconstitute the *A. suum* AChR, such that varying the amounts of the subunit cRNAs injected changed the pharmacology of the expressed receptors. This is similar to the *Ode(29–63)*, in that only two subunits were used to reconstitute a *Pyr-nAChR* and varying the amount of injected subunit cRNAs affected the pharmacology of the receptor. Whereas no ancillary factors were used to reconstitute the *A. suum* nAChRs, we observed that the three ancillary factors were required to reconstitute the *O. dentatum* receptors. Another observation is that the amounts of cRNA we injected to reconstitute the *O. dentatum* receptors were far less than what was injected to reconstitute the *A. suum* receptors. These demonstrate differences between the expressed receptors of nematodes in the same clade (*O. dentatum*, *C. elegans* and *H. contortus*) and/or in different clades (*O. dentatum* and *A. suum*). In addition we have also demonstrated tribendimidine's effect on all four *O. dentatum* receptor types and in two of the receptor types, the *EC_50_* values show tribendimidine was more potent than the other agonists across the types. We found that pyrantel was the most potent agonist of two types of *O. dentatum* receptors but it is only most potent in one of the types of *H. contortus*. The real and significant differences between the effects of pharmacological agents on the model nematode *C. elegans* and on the parasitic nematode *O. dentatum*, both Clade V nematodes, shows that species variation requires that effects of anthelmintics on the relevant nematode parasite are tested as a vital reality check.

## Materials and Methods

### Ethical concerns

All animal care and experimental procedures in this study were in strict accordance with guidelines of good animal practice defined by the Center France-Limousin ethical committee (France). The vertebrate animals (pig) studies were performed under the specific national (French) guidelines set out in the Charte Nationle portant sur l'ethique de l'experimentation animal of the Ministere De Lensiengnement Superieur et de la Recherch and Ministere De L'Agriculture et de la Peche experimental agreement 6623 approved by the Veterinary Services (Direction des Services Vétérinaires) of Indre et Loire (France).

### Nematode isolates

These studies were carried out on the levamisole-sensitive (SENS) isolate of *O. dentatum* as previously described [40]. Large pigs were experimentally infected with 1000 infective larvae (L3s) and infection was monitored 40 days later by fecal egg counts every 3 days. The pigs were slaughtered after 80 days at the French National Institute for Agricultural Research (Nouzilly) abattoir and adult nematodes (males and females) were collected from the large intestine and stored in RNA later (Qiagen®) at −80°C.

### Molecular biology

Ten adult males from *O. dentatum* SENS isolate were used for total RNA preparation using TRIzol (Invitrogen) according to the manufacturer's instructions. First-strand cDNA synthesis on resuspended and DNase-treated total RNA was carried out with the oligo (dT) RACER primer and superscript III reverse transcriptase (Invitrogen, Carlsbad, CA, USA) as previously described [16]. To identify full-length cDNA sequences of *O. dentatum unc-29*, *unc-63* and *acr-8* homologues, 5′- and 3′- rapid amplification of complementary ends (RACE) polymerase chain reactions (PCR) were performed using the splice leader sequence primer (SL1) with internal reverse primers and internal forward specific primers with reverse transcription 3′-site adapter primers, respectively. PCR products were cloned into the pGEM-T vector (Promega). Positive identifications were made by BLAST analysis against all *Haemonchus contortus* entries in the *H. contortus* information resource data base. Primers designed on *unc-29* sequence from *H. contortus* and *C. elegans* (unc-29-F0 GGACGAGAAAGATCAAGTTATGCA, unc-29-XR2 TCATCAAATGGGAAGAAYTCGACG) allowed PCR amplification of a 288 bp fragment corresponding to partial *O. dentatum unc-29* homologue. New gene specific primer sets were designed on this sequence for the 5′- and 3′-RACE-PCR providing the full-length cDNA sequence of *Ode-unc-29*. To identify the *unc-63* homologue from *O. dentatum*, degenerate primers were designed based on the *Ode-unc-38* mRNA sequence (accession number GU256648) and the *unc-63* and *unc-38* sequences of cDNAs available from multiple nematode species (Ode-unc-63-F1 GCGAATCGCGAYGCGAATCGKCT, F2 AARAGYATGTGYCAAATWGAYGT, R3 ATATCCCAYTCGACRCTGGGATA, R4 TAYTTCCAATCYTCRATSACCTG). An 861 bp partial coding sequence of *Ode-acr-8* cDNA was amplified using a sense primer (Hco-acr-8-FT1 TATGGTTAGAGATGCAATGGTT) and an antisense primer (Hco-acr-8-RE8 GTGTTTCGATGAAGACAGCTT) from *H. contortus Hco-acr-8*. The product of this reaction was cloned and sequenced, and the data were used to design primers to get the 5′ and the 3′ ends of the target transcript. To amplify the full-length coding sequence of *Ode-unc-38*, *Ode-unc-63*, *Ode-unc-29* and *Ode-acr-8*, we used the Phusion DNA polymerase (Finnzymes) and gene specific primer pairs containing *Hind*III or *Xho*I and *Apa*I restriction enzyme sites (Table S1 in Text S1) to facilitate directional cloning into the pTB-207 expression vector that is suitable for *in vitro* transcription. PCR products were then digested with XhoI and ApaI restriction enzymes (except *Ode-unc-29*, digested with HindIII and ApaI), purified using the NucleoSpin Gel and PCR Clean-up kit (Macherey-Nagel), ligated into the respective corresponding sites of the pTB-207 expression vector with T4 DNA Ligase (New England Biolabs), and resulting constructs were transformed into *E. coli* DG1 cells (Eurogentec) [26]. Three clones of each gene were sequenced using the standard primers T7 and polyT-V. Each of the reported sequences in supplementary figures S1A–D is from one of the nearly identical clones. The mMessage mMachine T7 transcription kit (Ambion) was used for *in vitro* cRNA synthesis from linearized plasmid DNA templates. The cRNAs were precipitated by lithium chloride, resuspended in RNAse-free water and stored at −80°C.

### Sequence analysis and accession numbers

Database searches, prediction of conserved motifs and phylogenetic analyses for the cDNAs were carried out as already described [16]. Maximum likelihood analysis was performed on full-length AChR subunit cDNA sequences as follows: sequences were aligned with Geneious 6.1.6 (Biomatters Ltd) using the translation align MAFFT plugin [41] that aligns the nucleotide sequences as codons based on amino acid sequences. The resulting nucleotide alignment was used to generate phylogenetic trees with the PhyML plugin for Geneious [42]. The HK85 substitution matrix was selected in order to optimize the tree topology and branch lengths. Branch support was evaluated using the chi2 option of PhyML. The accession numbers for cDNA and protein sequences mentioned in this article are *C. elegans*: UNC-29 NM_059998, UNC-38 NM_059071, UNC-63 NM_059132, ACR-8 JF416644; *H. contortus*: *Hco-unc-29.1* GU060980, *Hco-unc-38* GU060984, *Hco-unc-63a* GU060985, *Hco-acr-8* EU006785, *Hco-unc-50* HQ116822, *Hco-unc-74* HQ116821, *Hco-ric-3.1* HQ116823; *O. dentatum*: *Ode-unc-29* JX429919, *Ode-unc-38* JX429920, *Ode-unc-63* HQ162136, *Ode-acr-8* JX429921.

### Voltage-clamp studies in oocytes

*Xenopus laevis* ovaries were obtained from NASCO (Fort Atkinson, Wisconsin, USA) and defolliculated using 1–2 mg/ml collagenase type II and Ca^2+^-free OR2 (mM: NaCl 100, KCl 2.5, HEPES 5, pH 7.5 with NaOH). Alternatively, defolliculated oocytes were purchased from Ecocyte Bioscience (Austin, Texas, USA). Oocytes (animal pole) were microinjected with ∼36 nL of a cRNA injection mix containing 50 ng/µL of each subunit cRNA, as described in [26]. Equal amounts of the *H. contortus* ancillary factors *ric-3, unc-50* and *unc-74* were added to each mix. Microinjected oocytes were incubated at 19°C for 2–5 days. The oocytes were incubated in 200 µL of 100 µM BAPTA-AM for ∼3 hours prior to recordings, unless stated otherwise. In control experiments, we recorded from un-injected oocytes. Recording and incubation solutions used are reported in [26]. Incubation solution was supplemented with Na pyruvate 2.5 mM, penicillin 100 U/mL and streptomycin 100 µg/ml. All drugs applied, except tribendimidine and derquantel, were purchased from Sigma Aldrich (St Louis, MO, USA). Oocytes were voltage-clamped at −60 mV with an Axoclamp 2B amplifier; all data were acquired on a desktop computer with Clampex 9.2.

### Single-channel studies

Oocyte-attached (outside-out) patch-clamp procedure was used to obtain all recordings at room temperature (20°C–25°C). Oocytes were prepared for the recordings by removal of the vitelline membrane with forceps after placing in hypertonic solution, as already described [43]. The oocytes were transferred to the recording chamber and bathed in a high Cs solution with no added Ca^2+^ (composition in mM: CsCl 140, MgCl_2_ 2, HEPES 10, EGTA 1, pH 7.3) to reduce K^+^ currents and lower opening of Ca^2+^-activated Cl^−^ channels. Patch electrodes were pulled from thin-walled capillary glass tubing (Warner Instruments, Hamden, CT), coated close to the tip with sylgard and fire-polished. The electrodes were filled with a high pipette solution {composition in mM: CsCl 35, CsAc 105, CaCl_2_ 1, HEPES 10} containing 10 µM levamisole. Axopatch 200B amplifier (Axon Instruments, Union City, CA) was used to amplify currents which were sampled at 25 kHz with Digidata 1320A (Axon Instruments) and filtered at 1.5 kHz (3-pole Bessel). The linear least squares regression was used to estimate the conductances of the channels since the slopes were linear and did not show rectification.

### Data analysis

Acquired data were analyzed with Clampfit 9.2 (Molecular Devices, Sunnyvale, CA, USA) and Graphpad Prism 5.0 software (San Diego, CA, USA). The peak of currents in BAPTA-soaked oocytes was measured. The response to 100 µM ACh was normalized to 100% and the responses to the other agonists normalized to that of ACh. For all dose-response relationships, the mean ± s.e of the responses is plotted. Dose-response data points were fitted with the Hill equation as described previously [26]. To calculate the *pK_B_* values, the graphs were fitted with the Gaddum/Schild *EC_5_*_0_ shift and the bottom of the curves were set to zero.

## Supporting Information

Figure S1 A–D. Amino acid alignments of the four *O. dentatum* nAChR subunits with the *H. contortus* and *C. elegans* homologues. The sequences were aligned with the MUSCLE algorithm [44] and processed further with GeneDoc. Shaded in grey are the predicted signal peptides and in blue are the amino acids conserved between all three species. Noted above the aligned sequences are the cys-loop, Yx(x)CC motif and transmembrane domains. Noted with red arrows (S1 A–C) are the amino acids, on either side of the transmembrane region (TM2) that are predicted to contribute to the permeability of the channel to calcium [45], [46]. Green arrow in S1 D shows a histidine which implies pH sensitivity around pH 6.5 to channel permeability.
(TIF)

Figure S2 Distance tree showing relationships of nicotinic acetylcholine receptors (nAChR) subunit sequences in *Oesophagostomum dentatum* (Ode, highlighted in red), *Caenorhabditis elegans* (Cel), *Haemonchus contortus* (Hco) and *Teladorsagia circumcincta* (Tci). Numbers at each branch indicate percentage boostrap values corresponding to 1000 replicates. The scale bar represents substitutions per site. The *C. elegans acr-16* nAChR subunit was used as an outgroup. Distance analyses were performed on full-length cDNA sequences. Multiple alignment was performed using Muscle program with standard parameters [47]. Relationships between sequences were determined using the neighbor-joining method and the HKY substitution model [48]. One thousand bootstrap replicates were performed to test the support of nodes.
(TIF)

Figure S3 Effect of the ancillary proteins on receptor reconstitution. (A) Bar chart (mean ± se) of agonist-elicited currents in the *Ode*(*29–63–38–8*) or Lev-nAChR subtype. This receptor subtype was used to test the effect of sequentially removing the ancillary proteins on the reconstitution. All responses were normalized to control 100 µM ACh (unfilled bar) currents in oocytes expressing this receptor subtype with all the ancillary proteins (A). (B) Effect of removing *unc-74* from the mix on the *Ode*(*29–63–38–8)/*Lev-nAChR subtype. Note the relative change in ACh and Lev responses. (C) Effect of removing *ric-3* on the *Ode*(*29–63–38–8*) : Lev-nAChR subtype. The ACh current responses were reduced to <50% of the control. (D) Effect of removing *unc-50* on the *Ode*(*29–63–38–8*) or Lev-nAChR subtype. Note the dramatic decrease in currents elicited by all agonists.
(TIF)

Results S1 Supplementary results.
(DOCX)

Text S1 Supplementary information indicating how the calcium permeability measurements were conducted and the equation used for calculating the *P_Ca_/P_Na_* ratio. All assumptions for the permeability calculation are noted. For these set of experiments, we increased external Ca^2+^ in the recording solution from 1 mM to 10 mM without changing the concentration of the other ions. We used the GHK equation to calculate the permeability ratio, *P_Ca_/P_Na_*. Due to BAPTA treatment before recordings, we assumed the internal [Ca^2+^] to be negligible; we also assumed permeability to Na and K, *P_Na_* and *P_K_*, are equal. Ionic activities were used for the calculations (activity coefficients: 0.56 for Ca^2+^ and 0.72 for Na^+^ and K^+^). Below is the GHK equation used to calculate the calcium permeability ratio, with the activity coefficients for Na^+^, K^+^ and Ca^2+^ inserted: E_rev_ = RT/F In{(P_Na_*0.72[Na]_o_+P_K_*0.72[K]_o_+4P′*0.56[Ca]_o_)/{P_Na_*0.72[Na]_i_+PK*0.72[K]_i_)}. Where R is universal gas constant (8.314 JK^−1^mol^−1^); F is Faraday's constant (96485 Cmol^−1^); T is temperature (room temperature, 298 K); and *P′ = P_Ca_/P_Na_{1/(1+e^FErev/RT^)}*. A table of the primers (Table S1) used for the PCR and cloning of the four *O. dentatum* AChR subunits is also included here in Text S1.
(PDF)
