# Supplementary figures and images for: Investigation of Acetylcholine Receptor Diversity in a Nematode Parasite Leads to Characterization of Tribendimidine- and Derquantel-Sensitive nAChRs
Source: PLoS Pathog. 2014 Jan 30;10(1):e1003870. doi: 10.1371/journal.ppat.1003870 (PMC3907359; doi:10.1371/journal.ppat.1003870)

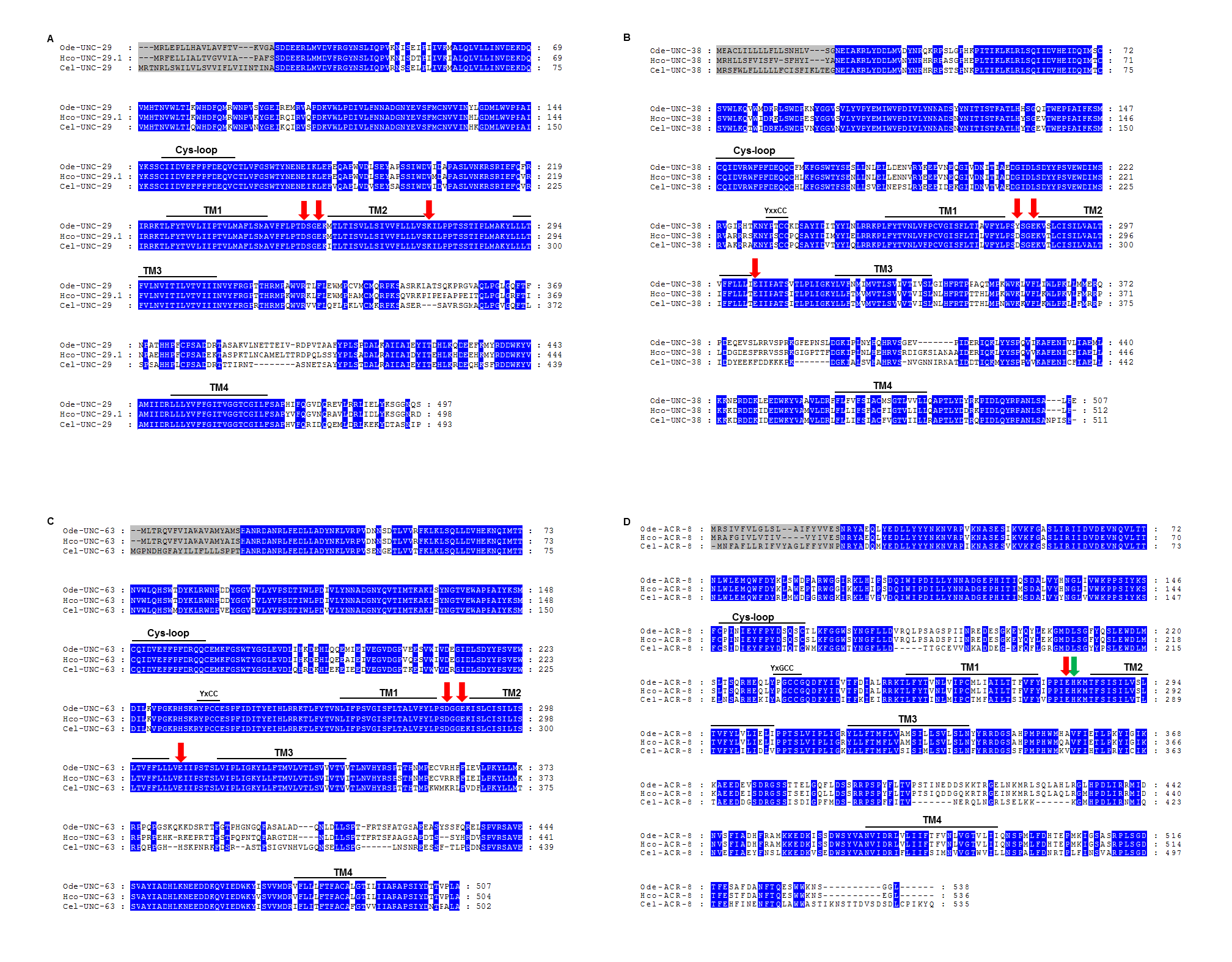

Supplement: Figure S1 — A–D. Amino acid alignments of the four O. dentatum nAChR subunits with the H. contortus and C. elegans homologues. The sequences were aligned with the MUSCLE algorithm [44] and processed further with GeneDoc. Shaded in grey are the predicted signal peptides and in blue are the amino acids conserved between all three species. Noted above the aligned sequences are the cys-loop, Yx(x)CC motif and transmembrane domains. Noted with red arrows (S1 A–C) are the amino acids, on either side of the transmembrane region (TM2) that are predicted to contribute to the permeability of the channel to calcium [45], [46]. Green arrow in S1 D shows a histidine which implies pH sensitivity around pH 6.5 to channel permeability. (TIF) [file ppat.1003870.s001.tif]

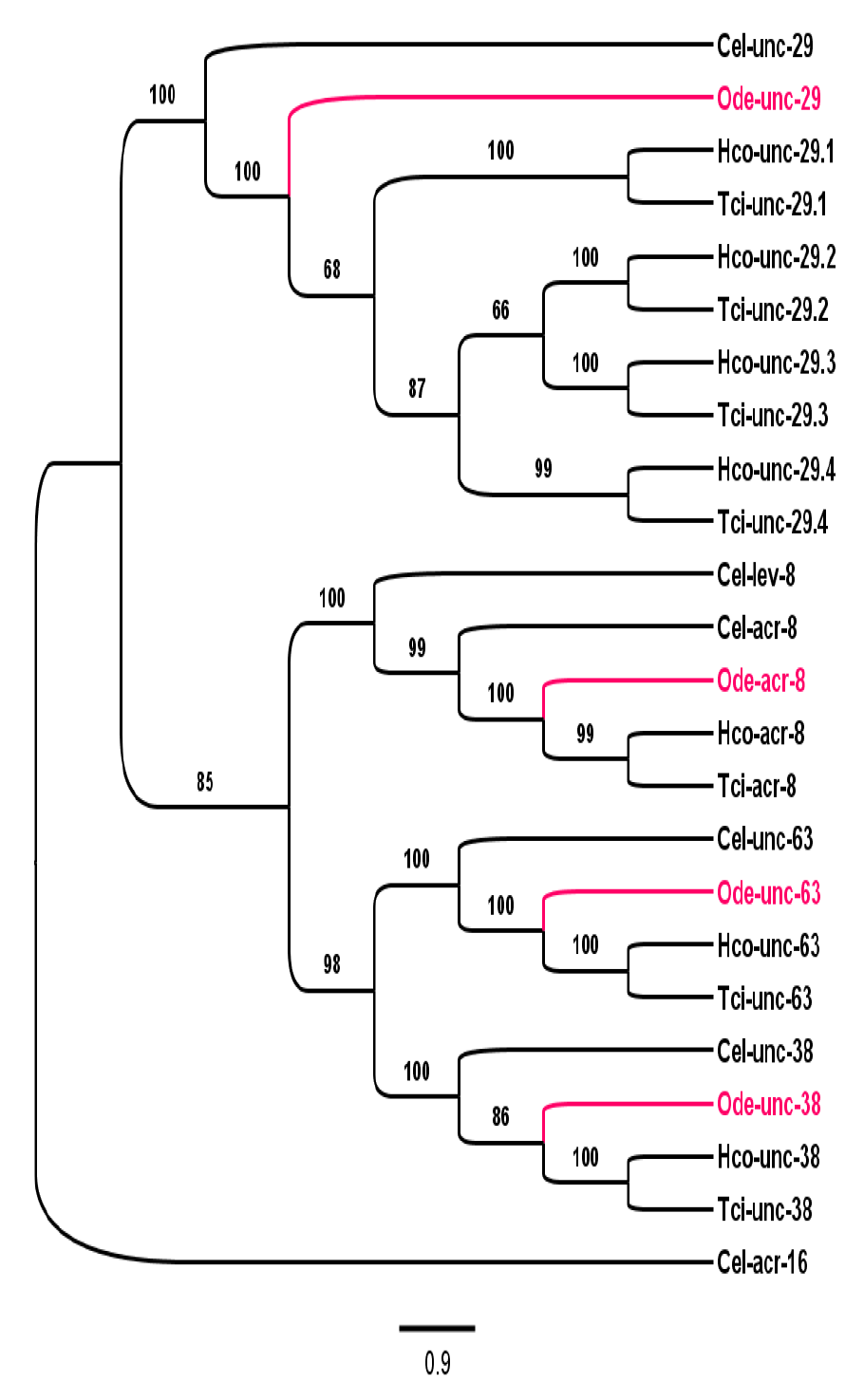

Supplement: Figure S2 — Distance tree showing relationships of nicotinic acetylcholine receptors (nAChR) subunit sequences in Oesophagostomum dentatum (Ode, highlighted in red), Caenorhabditis elegans (Cel), Haemonchus contortus (Hco) and Teladorsagia circumcincta (Tci). Numbers at each branch indicate percentage boostrap values corresponding to 1000 replicates. The scale bar represents substitutions per site. The C. elegans acr-16 nAChR subunit was used as an outgroup. Distance analyses were performed on full-length cDNA sequences. Multiple alignment was performed using Muscle program with standard parameters [47]. Relationships between sequences were determined using the neighbor-joining method and the HKY substitution model [48]. One thousand bootstrap replicates were performed to test the support of nodes. (TIF) [file ppat.1003870.s002.tif]

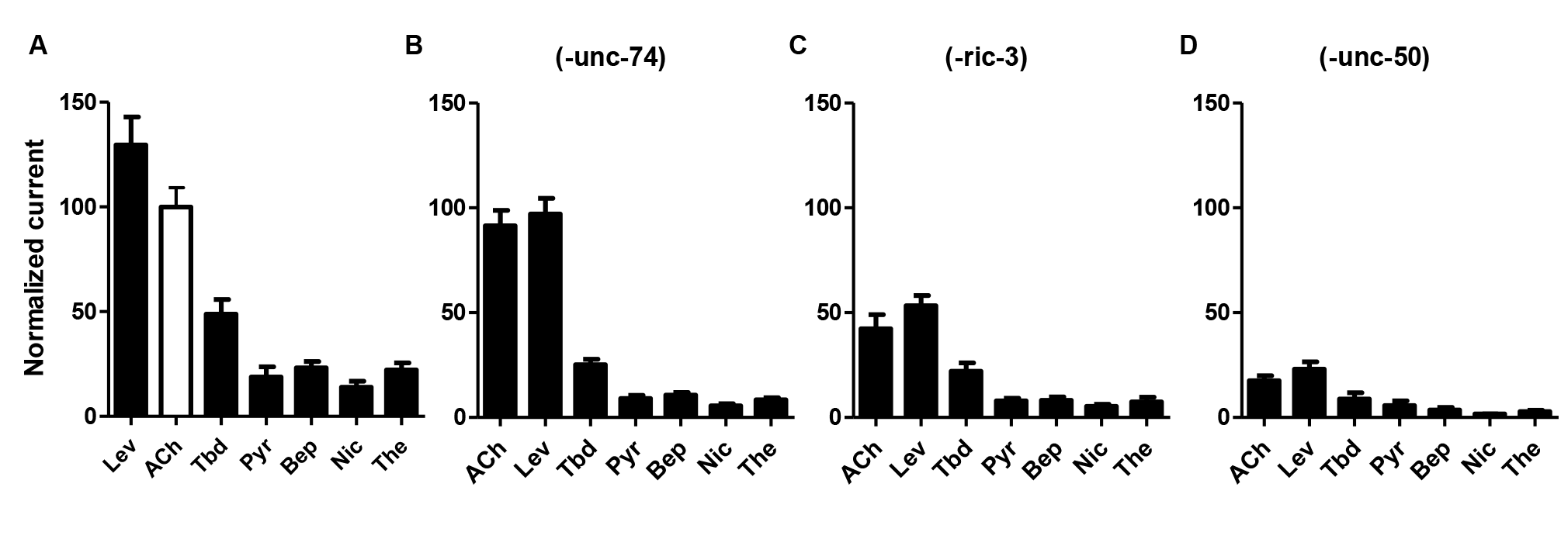

Supplement: Figure S3 — Effect of the ancillary proteins on receptor reconstitution. (A) Bar chart (mean ± se) of agonist-elicited currents in the Ode(29–63–38–8) or Lev-nAChR subtype. This receptor subtype was used to test the effect of sequentially removing the ancillary proteins on the reconstitution. All responses were normalized to control 100 µM ACh (unfilled bar) currents in oocytes expressing this receptor subtype with all the ancillary proteins (A). (B) Effect of removing unc-74 from the mix on the Ode(29–63–38–8)/Lev-nAChR subtype. Note the relative change in ACh and Lev responses. (C) Effect of removing ric-3 on the Ode(29–63–38–8) : Lev-nAChR subtype. The ACh current responses were reduced to <50% of the control. (D) Effect of removing unc-50 on the Ode(29–63–38–8) or Lev-nAChR subtype. Note the dramatic decrease in currents elicited by all agonists. (TIF) [file ppat.1003870.s003.tif]
