# Supplementary material for: Investigation of Acetylcholine Receptor Diversity in a Nematode Parasite Leads to Characterization of Tribendimidine- and Derquantel-Sensitive nAChRs
Source: PLoS Pathog. 2014 Jan 30;10(1):e1003870. doi: 10.1371/journal.ppat.1003870 (PMC3907359; doi:10.1371/journal.ppat.1003870)
Supplement: Results S1 — Supplementary results. (DOCX) [file ppat.1003870.s004.docx]

**Results S1. Supplementary Results.**

UNC-74 is a gene that encodes a transmembrane thioredoxin that can affect expression of nAChRs [26]. When we expressed the *Ode(29–63–8–38)* receptor with RIC-3 and UNC-50 but without UNC-74, the oocytes responded with acetylcholine currents that were only slightly reduced (mean 9%, S2-B). However, the order of the agonist potency series changed from 100 µM acetylcholine > 100 µM levamisole to 100 µM acetylcholine ≈ 100 µM levamisole (S2-A, B). We tested the agonist potency series of all the anthelmintic agonists and found that their normalized responses were significantly different (p< 0.0001, F-test). Thus, UNC-74 was not an absolute requirement for expression of *Ode*(*29–63–8–38)* but affected the pharmacological profile of the expressed receptors suggesting that it changed the balance of the receptor subtypes types present. RIC-3 is an endoplasmic reticulum resident protein that acts as a nAChR chaperone. RIC-3 enhances functional expression of some nAChRs, whilst inhibiting expression of other nAChRs [49,50,51]. RIC-3 stabilizes receptor intermediates and promotes maturation of receptors through subunit-specific interactions with nAChR subunits [52]. When we removed just RIC-3 from the mix, the acetylcholine and levamisole currents were reduced to < 60 % of control (S2-A, C). When we compared the acetylcholine-normalized responses of all the agonists we found that they were significantly different (p< 0.0001, F-test) suggesting that the balance of the receptor types present had changed.

The most striking decrease in current amplitude was obtained when just UNC-50 was removed. The acetylcholine current amplitudes obtained were < 20 % of control (S2-D). UNC-50 is a Golgi resident protein that is required to prevent some nAChR types from being trafficked to and degraded by the lysosomal system [53]. In the absence of UNC-50*,* it is reasonable to postulate that only a few synthesized receptors make it to the cell membrane. When we compared the acetylcholine-normalized responses of all the anthelmintic agonists to the control, we again found that they were significantly different (p< 0.0001, F-test), suggesting that the balance of the receptor types expressed was changed.
