# Supplementary material for: Investigation of Acetylcholine Receptor Diversity in a Nematode Parasite Leads to Characterization of Tribendimidine- and Derquantel-Sensitive nAChRs
Source: PLoS Pathog. 2014 Jan 30;10(1):e1003870. doi: 10.1371/journal.ppat.1003870 (PMC3907359; doi:10.1371/journal.ppat.1003870)
Supplement: Text S1 — Supplementary information indicating how the calcium permeability measurements were conducted and the equation used for calculating the PCa/PNa ratio. All assumptions for the permeability calculation are noted. For these set of experiments, we increased external Ca2+ in the recording solution from 1 mM to 10 mM without changing the concentration of the other ions. We used the GHK equation to calculate the permeability ratio, PCa/PNa. Due to BAPTA treatment before recordings, we assumed the internal [Ca2+] to be negligible; we also assumed permeability to Na and K, PNa and PK, are equal. Ionic activities were used for the calculations (activity coefficients: 0.56 for Ca2+ and 0.72 for Na+ and K+). Below is the GHK equation used to calculate the calcium permeability ratio, with the activity coefficients for Na+, K+ and Ca2+ inserted: Erev = RT/F In{(PNa*0.72[Na]o+PK*0.72[K]o+4P′*0.56[Ca]o)/{PNa*0.72[Na]i+PK*0.72[K]i)}. Where R is universal gas constant (8.314 JK−1mol−1); F is Faraday's constant (96485 Cmol−1); T is temperature (room temperature, 298 K); and P′ = PCa/PNa{1/(1+eFErev/RT)}. A table of the primers (Table S1) used for the PCR and cloning of the four O. dentatum AChR subunits is also included here in Text S1. (PDF) [file ppat.1003870.s005.pdf]

Supporting Information

Materials and Methods

Ca^{2+}-permeability measurements

For these set of experiments, we increased external Ca^{2+} in the recording solution from 1 mM to 10 mM without changing the concentration of the other ions. We used the GHK equation to calculate the permeability ratio, P_{Ca}/P_{Na}. Due to BAPTA treatment before recordings, we assumed the internal [Ca^{2+}] to be negligible; we also assumed permeability to Na and K, P_{Na} and P_K, are equal. Ionic activities were used for the calculations (activity coefficients: 0.56 for Ca^{2+} and 0.72 for Na^+ and K^+). Below is the GHK equation used to calculate the calcium permeability ratio, with the activity coefficients for Na^+, K^+ and Ca^{2+} inserted.

$$E_{rev} = RT/F\ In\{(P_{Na}*0.72[Na]_o+P_K*0.72[K]_o+4P'*0.56[Ca]_o)/\{P_{Na}*0.72[Na]_i+PK*0.72[K]_i)\}$$

Where R is the universal gas constant (8.314 $JK^{-1}mol^{-1}$); F is Faraday's constant (96485 $Cmol^{-1}$); T is the temperature (room temperature, 298 K); and $P' = P_{Ca}/P_{Na}\{1/(1+e^{FErev/RT})\}$.

Table S1.0 Primers used for the PCR and cloning of *O. dentatum* AChR subunits

**Gene name**	**Primer name**	**Primer sequence**
Ode-unc-29	F-Hind3	AAAAAGCTTATGCGTCTCGAACCGTTACTTC
	R-Apa1	TTTGGGCCCTAAACCCGTACAGTCATAAAACAAT
Ode-unc-38	F-Xho1	AAACTCGAGATAGCTGGTTGCAAGTGCGTATT
	R-Apa1	TTTGGGCCCTCTCAACAAAATTGGCCTAATATAC
Ode-unc-63	F-Xho1	AAACTCGAGATGCTGACGCGACAAGTGTTC
	R-Apa1	TTTGGGCCCTACCCAGCCGGCTGCTCGC
Ode-acr-8	F-Hind3	AAACTCGAGCTTGGCTAGCTTAAAACTAAGATT
	R-Apa1	TTTGGGCCCAACCATAATACTATACATATCTCAGA
